# The SaeRS Two-Component System Controls Survival of *Staphylococcus aureus* in Human Blood through Regulation of Coagulase

**DOI:** 10.3389/fcimb.2017.00204

**Published:** 2017-05-29

**Authors:** Haiyong Guo, Jeffrey W. Hall, Junshu Yang, Yinduo Ji

**Affiliations:** ^1^Department of Biological Science, School of Life Science, Jilin Normal UniversitySiping, China; ^2^Department of Veterinary and Biomedical Sciences, College of Veterinary Medicine, University of MinnesotaSt Paul, MN, United States

**Keywords:** *S*. *aureus*, survival, two-component system, SaeRS, coagulase

## Abstract

The SaeRS two-component system plays important roles in regulation of key virulence factors and pathogenicity. In this study, however, we found that the deletion mutation of *saeRS* enhanced bacterial survival in human blood, whereas complementation of the mutant with SaeRS returned survival to wild-type levels. Moreover, these phenomena were observed in different MRSA genetic background isolates, including HA-MRSA WCUH29, CA-MRSA 923, and MW2. To elucidate which gene(s) regulated by SaeRS contribute to the effect, we conducted a series of complementation studies with selected known SaeRS target genes *in trans*. We found coagulase complementation abolished the enhanced survival of the SaeRS mutant in human blood. The *coa* and *saeRS* deletion mutants exhibited a similar survival phenotype in blood. Intriguingly, heterologous expression of coagulase decreased survival of *S. epidermidis* in human blood. Further, the addition of recombinant coagulase to blood significantly decreased the survival of *S. aureus*. Further, analysis revealed staphylococcal resistance to killing by hydrogen peroxide was partially dependent on the presence or absence of coagulase. Furthermore, complementation with coagulase, but not SaeRS, returned *saeRS*/*coa* double mutant survival in blood to wild-type levels. These data indicate SaeRS modulates bacterial survival in blood in coagulase-dependent manner. Our results provide new insights into the role of staphylococcal SaeRS and coagulase on bacterial survival in human blood.

## Introduction

*Staphylococcus aureus* is an important pathogen that can cause various infections, including skin and soft tissue infection and systematic infections such as pneumoniae, endocarditis, and toxic shock syndrome (Klevens et al., 2007; Gordon and Lowy, 2008). The pathogenicity of *S. aureus* is attributable to its ability to produces different virulence factors, including a series of cell wall-associated proteins and a range of extracellular cytotoxins, proteases, DNases, and enterotoxins (Foster and Höök, 1998), which enable the bacteria to evade innate and or adaptive immune systems (Liu et al., 2005; Clauditz et al., 2006; Lacey et al., 2016) and induce disease.

Two-component signal regulatory systems (TCSs) collaborate with transcriptional regulators to regulate the expression of virulence factors, which in turn contribute to the pathogenesis of *S. aureus*. The well-studied global and TCSs virulence regulators include Agr (Novick, 2003; Montgomery et al., 2010; Thoendel and Horswill, 2013), ArlRS (Fournier et al., 2001; Bronner et al., 2004; Liang et al., 2005), SaeRS (Giraudo et al., 1999; Liang et al., 2006; Voyich et al., 2009; Nygaard et al., 2010; Zurek et al., 2014; Liu et al., 2016), SarA (Chien et al., 1999; Cheung et al., 2008; Li et al., 2016; Loughran et al., 2016), and MgrA (Ballal et al., 2009; Gupta et al., 2015).

The SaeRS TCS controls the expression of critical virulence genes. SaeRS up-regulates the transcription and expression of *hla, hlb, hlgABC, lukED*, and *coa in vitro* (Giraudo et al., 1999; Liang et al., 2006; Rogasch et al., 2006; Nygaard et al., 2010), as well as controls *hla* expression *in vivo*, as the *sae* null mutation significantly decreased the expression of α-toxin (*hla*) during infection (Goerke et al., 2001, 2005). The mutation of *sae* eliminated the expression of *fnbA*, but increased the expression of CP5 in *S. aureus* strain Newman (Steinhuber et al., 2003). As a consequence of SaeRS signaling pathway disruption, the cytotoxicity and ability of *S. aureus* to adhere to and invade epithelial cells (Liang et al., 2006) and endothelial cells (Steinhuber et al., 2003) is impaired. In addition, it has been demonstrated that the SaeRS system is an important virulence regulator in various animal models of infection (Goerke et al., 2005; Liang et al., 2006; Voyich et al., 2009; Montgomery et al., 2010; Cho et al., 2015; Zhao et al., 2015).

Staphylococcal coagulase is directly regulated by SaeRS (Liu et al., 2016) and is an important factor for distinguishing *S. aureus* and coagulase negative staphylococcus. Coagulase converts host prothrombin to staphylothrombin, leading to activate the protease activity of thrombin. It was predicted that coagulase could protect bacteria from phagocytic and immune defenses by causing localized clotting, however, there are contradictory reports regarding the role of coagulase in pathogenicity in animal models of infection (Baddour et al., 1994; Moreillon et al., 1995; Cheng et al., 2010).

In this study, we aimed to determine the role of SaeRS in survival capacity of *S. aureus* in human blood. We utilized two published SaeRS mutant strains, including hospital-acquired methicillin resistant *S. aureus* (HA-MRSA) WCUH29 and USA400 community-associated (CA)-MRSA MW2 isolates, generated a *saeRS* deletion mutant in a USA300 CA-MRSA 923 human isolate, and examined the impact of the *saeRS* mutation on bacterial survival in the blood. Using targeted complementation studies, gene deletions, and purification of recombinant proteins, we determined the SaeRS regulated *coa* gene, encoding coagulase, was a mediator of enhanced *saeRS* mutant survival in human blood. Hence, modulation of *coa* expression by SaeRS may contribute to *S. aureus* survival in human blood and bacteremia.

## Materials and methods

### Bacterial strains, plasmids, and growth media

The bacterial strains and plasmids used in this study are listed in Table 1. *Escherichia coli* DC10B (gift of T.J. Foster) served as the host for all *in vitro* recombinant DNA (Monk et al., 2012). *E. coli* transformants were selected on Brain Heart Infusion (BHI; Difco) agar containing erythromycin (100 μg/ml) or Luria-Bertani agar containing ampicillin (100 μg/ml). *S. aureus* was cultured in Trypticase Soy Broth (TSB; Difco) or on TSA agar at 37°C with appropriate antibiotics. All bacterial cell cultures were incubated with shaking at 220 RPM. *S. aureus* transformants were selected on TSA containing chloramphenicol (10 μg/ml) or erythromycin (5 μg/ml).

**Table 1:** **Bacterial strains and plasmids used in this study**.

**Strain or plasmid**	**Relevant characteristics**	**References**
DC10B	Dam*^−^ E. coli*	Monk et al., 2012
BL21	Recombinant protein expression strain *E. coli*	
WCUH29	Human clinical MRSA isolate, *sigB*^+^/*rsbU*^+^	NCIMB40771; Hall et al., 2017
WCUH29/pYH4	WCUH29 with empty pYH4; Erm^R^	Sun et al., 2005
Sa371	WCUH29 *saeS* allelic replacement mutant with *tetA* gene; Tc^R^	Liang et al., 2006
Sa371/pYH4	WCUH29 *saeS::tetA* with pYH4; Tc^R^, Erm^R^	Liang et al., 2006
Sa371com	WCUH29 *saeS::tetA* with pYH4-*saeS*; Tc^R^, Erm^R^	Liang et al., 2006
Sa371/pYH4-*sa1000*	WCUH29 *saeS*::*tetA* with pYH4-*sa1000*; Tc^R^, Erm^R^	This study
Sa371/pYH4-*efb*	WCUH29 *saeS*::*tetA* with pYH4-*efb*; Tc^R^, Erm^R^	This study
Sa371/pYH4-*fnbAB*	WCUH29 *saeS*::*tetA* with pYH4-*fnbAB*; Tc^R^, Erm^R^	This study
Sa371/pYH4-*coa*	WCUH29 *saeS*::*tetA* with pYH4-*coa*; Tc^R^, Erm^R^	This study
WCUH29Δ*coa*	WCUH29 *coa* deletion mutant	This study
Sa371Δ*coa*	WCUH29 *saeS* and *coa* double mutant; Tc^R^	This study
WCUH29Δ*coa*/pYH4	WCUH29 *coa* deletion mutant with pYH4; Erm^R^	This study
WCUH29Δ*coa*/pYH4-*coa*	WCUH29 *coa* deletion mutant with pYH4-*coa*; Erm^R^	This study
Sa371Δ*coa*/pYH4	WCUH29 *coa* deletion mutant with pYH4; Tc^R^, Erm^R^	This study
Sa371Δ*coa*/pYH4-*coa*	WCUH29 s*aeR*S and *coa* double mutant with pYH4-*coa*; Tc^R^, Erm^R^	This study
Sa371Δ*coa*/pYH4-*saeRS*	WCUH29 *saeRS* and *coa* double mutant with pYH4-*saeRS*; Tc^R^, Erm^R^	This study
923	USA300 CA-MRSA	Montgomery et al., 2010
923Δ*saeRS*	923 *saeRS* deletion mutant	This study
923Δ*coa*	923 *coa* deletion mutant	This study
923Δ*saeRS*Δ*coa*	923 *saeRS* and *coa* double deletion mutant	This study
923/pYH4	923 with pYH4; Erm^R^	This study
923Δ*saeRS*/pYH4	923 *saeRS* deletion mutant with pYH4; Erm^R^	This study
923Δ*saeR*S/pYH4-*saeRS*	923 *saeRS* deletion mutant with pYH4-*saeRS*; Erm^R^	This study
923Δ*coa*/pYH4	923 *coa* deletion mutant with pYH4; Erm^R^	This study
923Δ*coa*/pYH4-*coa*	923 *coa* deletion mutant with pYH4-coa; Erm^R^	This study
923Δ*saeRS*Δ*coa*/pYH4	923 *saeRS* and *coa* double deletion mutant with pYH4; Erm^R^	This study
923Δ*saeRS*Δ*coa*/pYH4-*coa*	923 *saeRS* and *coa* double deletion mutant with pYH4-*coa*; Erm^R^	This study
923Δ*saeRS*Δ*coa*/pYH4-*saeRS*	923 *saeRS* and *coa* double deletion mutant with pYH4-*saeRS*; Erm^R^	This study
MW2	USA400 CA-MRSA human clinical isolate	Herold et al., 1998
NW2*saeRS*::*spec*	MW2 *saeRS* allelic replacement with spectinomycin resistant gene; Spec^R^	Voyich et al., 2009
NW2*saeRS*::*spec*/pYH4	NW2 *saeRS*::*spec* with pYH4; Erm^R^, Spec^R^	This study
NW2*saeRS*::*spec*/pYH4-*saeRS*	NW2 *saeRS*::*spec* with pYH4-*saeRS*; Erm^R^, Spec^R^	This study
MW2Δ*coa*	MW2 *coa* deletion mutant	This study
MW2*saeRS*::*spec*Δ*coa*	MW2 *saeRS* and *coa* double mutant; Spec^R^	This study
MW2/pYH4	MW2 with pYH4; Erm^R^	This study
MW2Δ*coa*/pYH4	MW2 *coa* deletion mutant with pYH4; Erm^R^	This study
MW2Δ*coa*/pYH4-*coa*	MW2 *coa* deletion mutant with pYH4-*coa*	This study
MW2*saeRS*::*spec*Δ*coa/*pYH4	MW2 *saeRS* and *coa* double mutant with pYH4; Erm^R^, Spec^R^	This study
MW2*saeRS*::*spec*Δcoa/pYH4-*coa*	MW2 *saeRS* and *coa* double mutant with pYH4-*coa*; Erm^R^, Spec^R^	This study
MW2*saeRS*::*spec*Δ*coa*/pYH4-*saeRS*	MW2 *saeRS* and *coa* double mutant with pYH4-*saeRS*, Erm^R^, Spec^R^	This study
*S. epidermidis*	Coagulase negative	Microbiology teaching lab
*S. epidermidis*/pYH4	*S. epidermidis* with pYH4; Erm^R^	This study
*S. epidermidis/*pYH4-*coa*	*S. epidermidis* with pYH4-*coa*; Erm^R^	This study
**PLASMIDS**		
pYH4	Shuttle vector with Tc inducible promoter; Erm^R^	Huang et al., 2004
pYH4-*sa1000*	*sa1000* cloned downstream of pYH4 tet promoter; Erm^R^	Liang et al., 2006
pYH4-*efb*	*efb*(*sa1003*) cloned downstream of pYH4 tet promoter; Erm^R^	Liang et al., 2006
pYH4-*coa*	*coa* cloned downstream of pYH4 tet promoter; Erm^R^	This study
pYH4-*fnbAB*	*fnbAB* cloned downstream of pYH4 tet promoter; Erm^R^	This study
pYH4-*saeS*	*saeS* cloned downstream of pYH4 tet promoter; Erm^R^	Liang et al., 2006
pYH4-*saeRS*	*saeRS* cloned downstream of pYH4 tet promoter; Erm^R^	This study
pKOR1	Temperature sensitive inducible allelic exchange plasmid for *S. aureus*; Cm^R^	Bae and Schneewind, 2006
pKOR1-*coa*	pKOR1 with in-frame *coa* upstream/downstream deletion region; Cm^R^	This study
pKOR1-*saeRS*	pKOR1 with in-frame *saeRS* upstream/downstream deletion region; Cm^R^	This study
**pET24b**		
pET24b-*sarZ*	*saZ* cloned his-tag expression vector PET24b	Liang et al., 2011
pET24b-*coa*	*coa* cloned his-tag expression vector PET24b	This study

### Construction of the *saeRS* and *coa* gene deletion mutants, and the *saeRS* and *coa* gene complemented strains

Deletion of *saeRS* and/*or coa* was carried out following the pKOR1 allelic exchange protocol as described (Sun et al., 2005; Bae and Schneewind, 2006) and primers sets saeRS-pKOR1 L–For/Rev and coa-pKOR1-R For/Rev listed in Table 2. The R-For primer was synthesized with a 5′ phosphate group. Each PCR fragment was purified and the two fragments were ligated together with T4 DNA ligase (Promega). The ligation product was mixed with BP Clonase, per manufacturer's instructions, and plasmid pKOR1, incubated at 25°C overnight, then transformed into *E. coli* DC10B. The pKOR1-*saeRS*KO or pKOR1-*coa*KO plasmid was subsequently transformed into *S. aureus* 923, WCUH29, or MW2, respectively. Big colonies were re-streaked to fresh TSA plates and deletion of *saeRS* and/or *coa* was confirmed by diagnostic PCR.

**Table 2:** **Oligonucleotides used in this study**.

**Primer**	**Sequence (5′–3′)**
saeRS-KO-pKOR1-L-F	CACGATCAGTAAGTGGGTCAT
saeRS-KO-pKOR1-L-R	GTAACATTACACAAATTAGACATTACGTCATAATC
saeRS-KO-pKOR1-R-F	GGGGACCACTTTGTACAAGAAAGCTGG GTGATGATGGAAGTACGGATACCAC
saeRS-KO-pKOR1-R-R	CGCACTCGAGTGACGTAATGTCTAATTTGTG
saeRfor1	GGGGACAAGTTTGTACAAAAAAGCAGGCTCGGTAAAGAAATCGCAATGGTTG
saeSrev	AAACTATGACCCACTTACTGATCG
saeSfor1	AAGCTAGCATGGTGTTATCAATTAGAAGTCAAATC
CoaRFor	ATTTATAACTCTATCCAAAGACATACAGTCAATAC
CoaRRevAttB2	GGGGACCACTTTGTACAAGAAAGCTGGGTGCCTATGACGCACAACGTGATGGTCG
CoaLForAttB1	GGGGACAAGTTTGTACAAAAAAGCAGGCTACATGAAGCAAAACTTGTATCCTTAGACG
CoaLRev	AATTTTTTAATTCCTCCAAAATGTAATTGCCC
Sa0222-for	GTTGTGTTGTTTCTTCAGCTTTACCAG
Sa0222-rev	CATGCCACACCATATTCTTCTCC
Coa-For-RBS	AGGAGGTTTAAACTATGAAAAAGCAAATAATTTCGCTAGGC
Coa-Rev-AscI	TTGGCGCGCCTTATTTTGTTACTCTAGGCCCATATGTCG
coaBamHIFor	TTTGGATCCATGAAAAAGCAAATAATTTCGCTAG
coaXholRev	CCGCTCGAGTTTTGTTACTCTAGGCCCATATG
FnbAB-For-RBS	AGGAGGTTTAAACT GTGAAAAACAATCTTAGGTACGGC
FnbAB-Rev-AscI	TTGGCGCGCCTTATGCTTTGTTATTCTTTTTATTTCTGCG
T7promoterfor	GATCTAAGCTTCGGGAATTCACTA G
T7terminatorrev	CAATACAATGTAGGCTGC

In order to examine whether the expression of *saeRS, coa in trans* can complement the effect of the mutation of the respective endogenous gene, we constructed recombinant plasmids, including pYH4/*saeRS*, pYH4/*sa1000*, pYH4/*efb*, pYH4/*coa* by cloning the *saeRS, sa1000, efb, fnbAB*, or *coa* coding region (which was obtained by PCR) into the *Asc*I and *Pme*I sites of pYH4 (Huang et al., 2004), and electroporated it into the *saeRS* and/or *coa* knockout mutant, respectively, resulting in Sa371com, Sa371/pYH4-*sa1000*, Sa371/pYH4-*efb*, Sa371/pYH4-*fnbAB*, Sa371/pYH4-*coa*, and *coa* complementation strains in Table 1. The recombinant plasmid DNA were isolated from the complementary strains and confirmed by PCR and DNA sequencing (data not shown).

### Cloning, expression, and purification of coagulase-his tagged fusion protein in *Escherichia coli*

The *coa* gene was obtained by PCR amplification using the primers listed in Table 2, cloned into pET24b, and resulted in pET24b-*coa*. Plasmid pET24b-*coa* was introduced into a BL21(DE3) strain. The resulting strain was grown in LB medium at room temperature; the expression of coagulase was induced when the culture media reached OD600 nm equal to 0.6 by addition of 1 mM IPTG (isopropyl-b-D-thiogalactoside) and incubation pursued for 4 h. The coagulase-his tagged protein was purified using Ni-NTA agarose column (Novagen) and examined using 12% SDS-PAGE and Coomassie Blue staining as described (Yang et al., 2015). The purification of SarZ-6 × His was carried out as described using the previously constructed pET24b-*sarZ* plasmid (Liang et al., 2011).

### Blood survival assay

Strains were cultured in TSB with appropriate antibiotics. Inducer anhydrotetracycline (ATc) was added when indicated to overnight cultures. Following 18 h of culturing, the bacteria were washed twice in sterile PBS and suspended to an OD of 0.14 using a Behring photometer in PBS. Fresh venous human whole blood was collected using heparin containing Vacutainer tubes (BD) from outwardly healthy adult donors. The blood was then immediately used in the assay as described (Liu et al., 2005; Hall et al., 2015, 2017). The percentage of surviving bacteria was calculated as (CFU_timepoint_/CFU_initialinput_)^*^100. Human blood collection was approved by the University of Minnesota Institutional Review Board.

### Hydrogen peroxide survival assay

To determine the contribution of coagulase to *S. aureus* survival when challenged with oxidative stress, overnight cultures were washed with PBS and ~2 × 10^8^ CFU were incubated at 37°C in a 1.5% hydrogen peroxide (H_2_O_2_)/PBS solution for 60 min (Liu et al., 2005). Serial dilutions were plated on TSA for enumeration of surviving CFU. Percent survival was calculated as surviving CFU/ input CFU multiplied by 100, (#CFU_f_/#CFU_i_)^*^100.

### Data analysis

Independent samples were statistically analyzed using a Student's *t-*test with an alpha level ≤ 0.05 considered significant. For data figures with more than two independent samples, a one-way ANOVA analysis with a *post-hoc* Tukey HSD-test was used to determine if there was statistical significance between samples with an alpha level ≤ 0.05 considered significant.

## Results

### The deletion mutation of *saeRS* enhanced *S. aureus* survival in human blood

**Figure 1:**
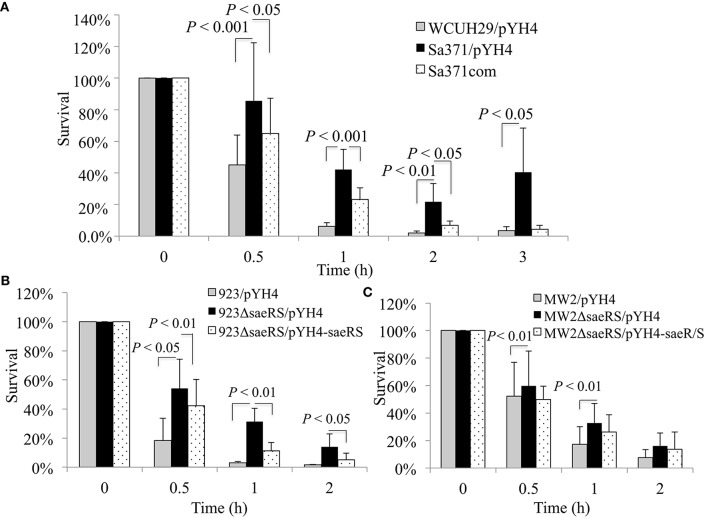
**Effect of ***saeS*** or ***saeRS*** null mutation and complementation on survival of ***S. aureus*** in human blood**. Percent survival of wild-type *S. aureus* HA-MRSA WCUH29 **(A)**, USA300 CA-MRSA 923 **(B)**, USA400 CA-MRSA MW2 **(C)**, and *saeRS* null mutants in freshly collected heparinized human blood with appropriate antibiotics. Bacteria were cultured overnight, diluted, inoculated into blood, and incubated at 37°C in a rotisserie incubator. Percent survival = (#CFU_final_/#CFU_input_)^*^100. The data represents the mean ± SEM of at least six independent experiments.

Our previous studies have demonstrated that the AirSR(YhcSR) system contributes *S. aureus* survival in human blood (Hall et al., 2015, 2017); and our preliminary study found that AirSR probably positively regulates the transcription of *saeRS* (data not shown). It has been well-documented that the SaeRS system is a key regulator of virulence factors that contribute to evade innate immune system and pathogenicity in a variety of animal models of *S. aureus* infection (Steinhuber et al., 2003; Liang et al., 2006; Zurek et al., 2014; Cho et al., 2015). These data led us to speculate that SaeRS system is likely involved in the function of AirSR in survival in the blood. To test this possibility, we deleted *saeS* and examined the impact of the deletion on bacterial survival in human blood. Unexpectedly, we found that the null mutation of *saeS* in HA-MRSA WCUH29 significantly increased survival in the blood throughout the 3 h experiment (Figure 1A).

To determine whether the genetic backgrounds of *S. aureus* affect SaeRS's mediated bacterial survival in the blood, we created a *saeRS* deletion mutant in a USA300 CA-MRSA 923 strain and acquired a *saeRS* allelic replacement mutant of USA400 CA-MRSA MW2 (Voyich et al., 2009). Consistent with the results of WCUH29, the *saeRS* null mutation remarkably increased the percentage of CFUs that survived in the blood for all strains (Figures 1B,C). Moreover, *in trans* SaeRS complemented strains had similar survival percentages as the wild-type controls (Figures 1A,B), indicating a specific effect of SaeRS on bacterial survival in human blood.

### The introduction of constitutive coagulase expression system eliminated the effect of the *saeRS* null mutation on bacterial survival in human blood

**Figure 2:**
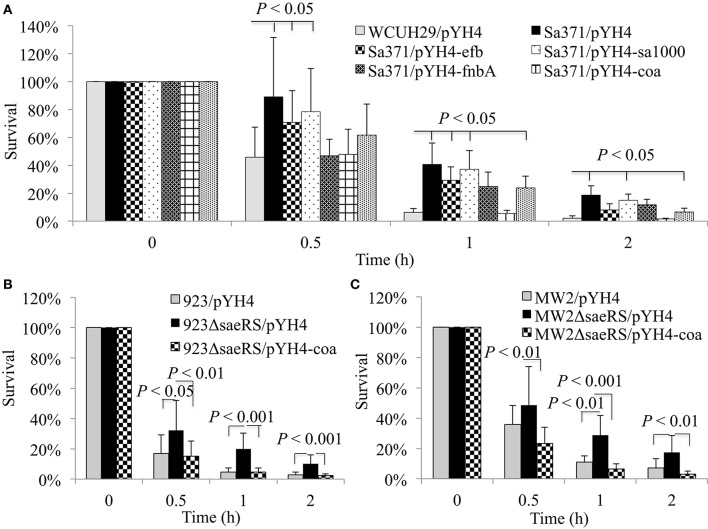
**Impact of ***sa1000***, ***efb***, ***fnbAB***, or ***coa*** expression ***in trans*** on enhanced survival of ***saeRS*** null mutants**. Percent survival of HA-MRSA WCUH29 **(A)**, USA300 CA-MRSA 923 **(B)**, USA400 CA-MRSA MW2 **(C)**, and the *sa1000, efb, fnbAB*, or *coa expression strain, and saeRS* complementation strain in freshly collected heparinized human blood with appropriate antibiotics. Data is the mean and standard error of at least six experiments per strain.

Previous studies revealed that the SaeRS system is a critical positive regulator of important virulence factors, including fibronectin-binding proteins (*fnbB, fnb*), fibrinogen-binding proteins (*efb*), coagulase (*coa*), and toxins (*hla, hlb*; Giraudo et al., 1999; Liang et al., 2006; Sun et al., 2010). We hypothesized the deletion of SaeRS prevented the expression of virulence factor with the result being increased bacterial survival in blood. In order to identify which SaeRS regulated gene(s) are involved in this phenomena, we tested the impact of *fnbAB, efb, sa1000*, or *coa* overexpression *in trans* on bacterial survival in blood in the *saeRS* mutants. The constitutive expression of coagulase restored the percentage of bacteria that survived to wild-type control levels and decreased the survival capacity of *S. aureus* compared to the *saeRS* null mutant control (Figures 2A–C). In contrast, the overexpression of FnbAB, Efb, or SA1000 did not eliminate the enhanced survival phenomena of the *saeRS* knockout mutant (Figure 2A).

### The deletion mutation of *coa* increased the survival of *S. aureus* in human blood

**Figure 3:**
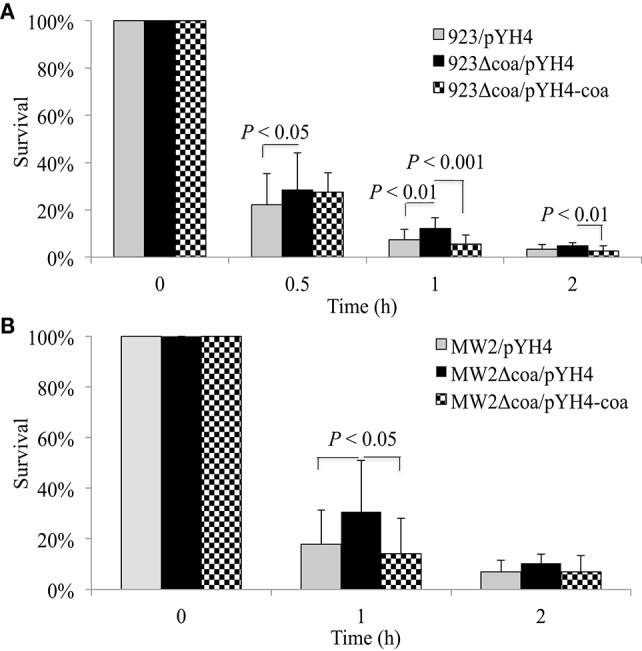
**Effect of ***coa*** deletion mutation and complementation on survival of ***S. aureus*** in human blood**. Percent survival of wild-type *S. aureus* USA300 CA-MRSA 923 **(A)**, USA400 CA-MRSA MW2 **(B)**, and *coa* deletion mutants and its complementation in freshly collected heparinized human blood with appropriate antibiotics. Bacteria were cultured overnight, diluted, inoculated into blood, and incubated at 37°C in a rotisserie incubator. Percent survival = (#CFU_final_/#CFU_input_)^*^100. The data represents the mean ± SEM of at least six independent experiments.

To confirm the role of coagulase in SaeRS's involvement in bacterial survival in the blood, we created *coa* deletion mutants in WCUH29, 923, and MW2 strains. Each new *coa* mutant was complemented and we tested each strain's ability to coagulate rabbit plasma. The *coa* deletion mutants of 923 and MW2 strains exhibited negative coagulation, whereas the *coa* deletion mutant of WCUH29 strain formed partial coagulation (Table 3). The 923 and MW2 wild type, *coa* null, and complemented strains were examined in our survival assays using human blood. Similar to the results of *saeRS* null mutants, the deletion of *coa* enhanced the survival capacity of *S. aureus* 923 and MW2 after 1 h of infection (Figures 3A,B). The *in trans coa* complementation restored the survival level to the wild-type control (Figures 3A,B), indicating coagulase production in blood is detrimental to the survival of *S. aureus*.

**Table 3:** **Coagulase activity of wild type strains, ***saeRS*** or ***coa*** null mutants, and ***saeRS*** or ***coa*** complemented strains**.

**Strains**	**Coagulation**	**Strains**	**Coagulation**
WCUH29	+	923	+
WCUH29Δ*coa*	±	923Δ*coa*	–
Sa371Δ*coa*	–	923Δ*saeRS*Δ*coa*	–
WCUH29/pYH_4_	+	923/pYH_4_	+
WCUH29Δ*coa*/pYH_4_	±	923Δ*coa*/pYH_4_	–
WCUH29Δ*coa*/pYH_4_-*coa*	+	923Δ*coa*/pYH_4_-*coa*	+
Sa371/pYH_4_	–	923Δ*saeRS*/pYH_4_	–
Sa371Δ*coa*/pYH_4_	–	923Δ*saeRS* Δ*coa*/pYH_4_	–
Sa371Δ*coa*/pYH_4_-*coa*	+	923Δ*saeRS* Δ*coa*/pYH_4_-*coa*	+
Sa371Δ*coa*/pYH_4_-*saeRS*	±	923Δ*saeRS* Δ*coa*/pYH_4_-*saeRS*	–
MW2	+	MW2Δ*coa*/pYH_4_-*coa*	+
MW2 Δ*coa*	–	MW2Δ*saeRS*/pYH_4_	–
MW2 Δ*saeRS*Δ*coa*	–	MW2Δ*saeRS* Δ*coa*/pYH_4_	–
MW2 /pYH_4_	+	MW2Δ*saeRS* Δ*coa*/pYH_4_-*coa*	+
MW2 Δ*coa*/pYH_4_	–	MW2Δ*saeRS* Δ*coa*/pYH_4_-*saeRS*	–

### The heterologous expression of coagulase or addition of recombinant coagulase inhibited survival ability in human blood

**Figure 4:**
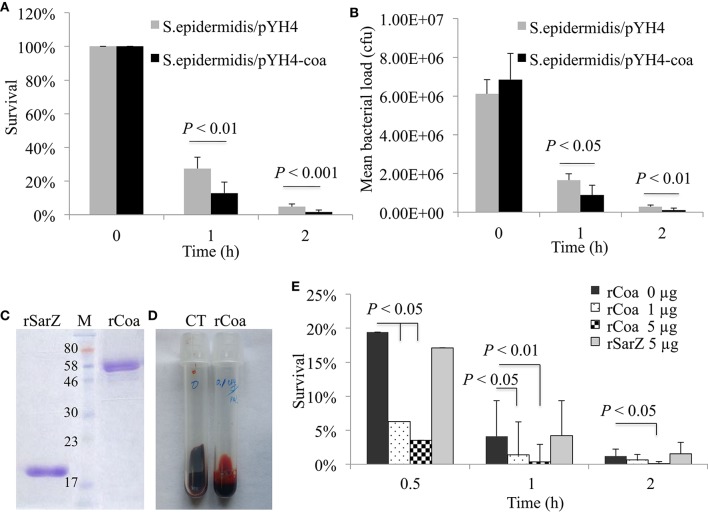
**Effect of ***coa*** heteroexpression or addition of recombinant coagulase on bacterial survival. (A)** Percent survival and **(B)** bacterial load of wild-type *S. epidermidis* and *coa* expression *in trans* in human blood during induction. **(C)** SDS-PAGE analysis of purified recombinant Coa (coagulase) and control protein SarZ from *E. coli*. M, ColorPlus Prestained protein Marker (NEB), the unit of size is kDa. **(D)** Coagulation activity of purified recombinant Coa (rCoa) from *E. coli* in human blood. CT, negative control without addition of rCoa. **(E)** Percent survival of wild-type USA300 CA-MRSA 923 and addition of different concentrations of purified rCoa and control rSarZ protein in human blood. Data is the mean and standard error of at least four experiments per strain.

To further confirm the role of coagulase in survival, we determined the effect of heterologous expressing coagulase in *S. epidermidis* on survival in the blood. Compared with the control, the induction of *coa* expression significantly decreased the survival of *S. epidermidis* in the blood throughout the duration of the experiment (Figures 4A,B). To define the role of coagulase in survival of *S. aureus*, we cloned, expressed, and purified recombinant coagulase (rCoa). The purity of purified rCoa was examined using SDS-PAGE (Figure 4C); the activity of purified rCoa was confirmed in a coagulation assay using human blood (Figure 4D). The addition of rCoa significantly decreased the survival ability of USA300 CA-MRSA 923 in human blood throughout the period of the experiment in a dose-dependent manner (Figure 4E). In contrast, the addition of control protein, recombinant SarZ, had no impact on survival compared to the negative control (Figure 4E).

### Coagulase is associated with susceptibility to hydrogen peroxide killing

**Figure 5:**
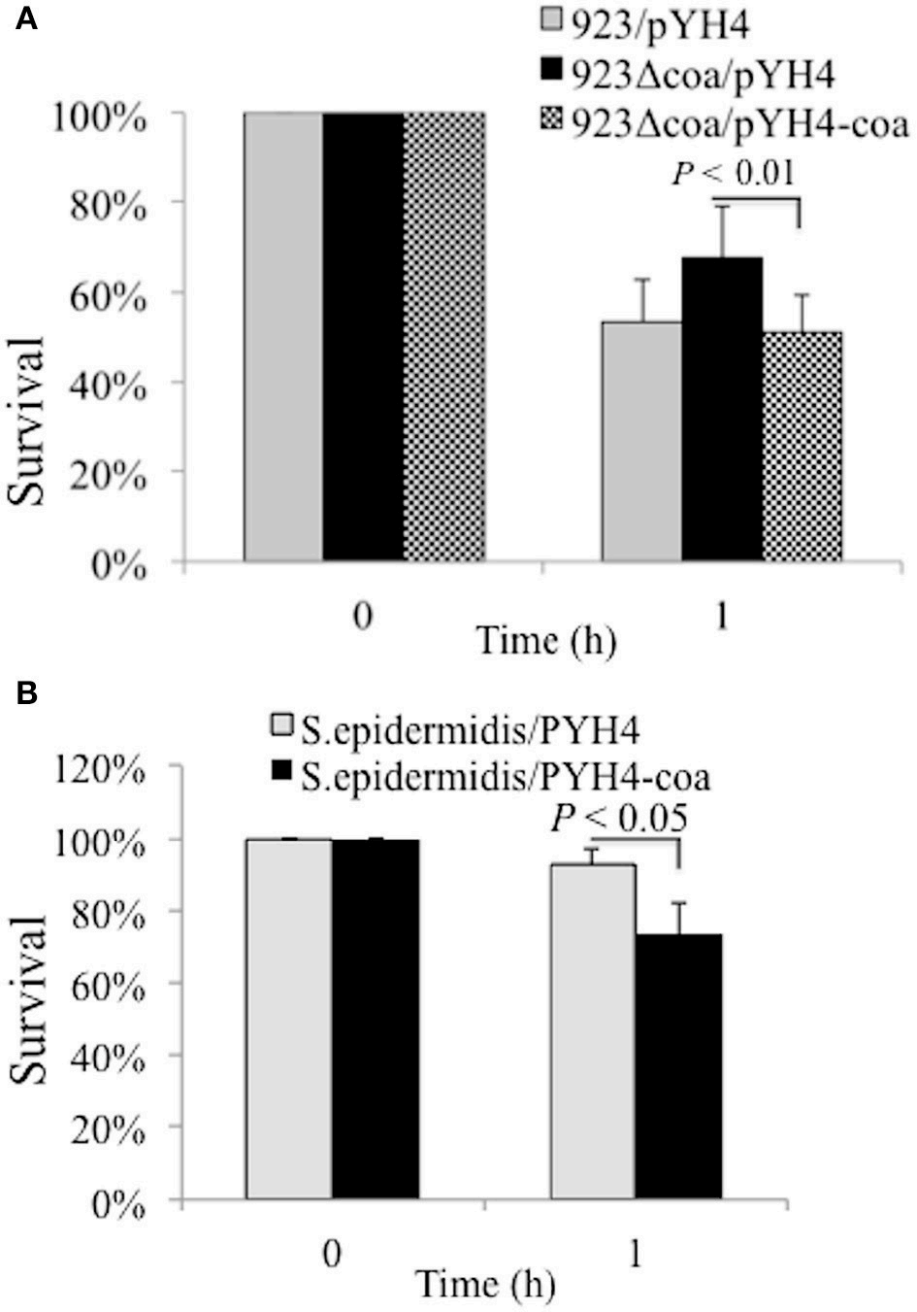
**Effect of Coa on bacterial resistance to H_**2**_O_**2**_. (A)** Percent survival of wild-type USA300 CA-MRSA 923, *coa* deletion mutant and complementation strain after exposure of H_2_O_2_. **(B)** Percent survival of wild-type *S. epidermiditis* and *coa* expression strain *in trans* after exposure of H_2_O_2_. The bacterial strains were cultured with appropriate antibiotics. Approximately 5 × 10^8^ CFU were incubated in sterile PBS with 1.5% hydrogen peroxide at 37°C for 1 h. The percent survival was calculated as (#CFU_final_/#CFU_input_)^*^100. The data represents the mean ± SEM of at least four independent experiments.

Coagulase is able to bind host prothrombin to form staphylothrombin, which in turn activates the protease activity of thrombin. Although it has been predicted that coagulase could protect bacteria from phagocytic and immune defenses by causing localized clotting, the role of coagulase in pathogenicity is contradictory (Baddour et al., 1994; Moreillon et al., 1995; Cheng et al., 2010). Reactive oxygen species (ROS) is a key element used by phagocytic cells to kill phagocytosed bacteria (Liu et al., 2005; Clauditz et al., 2006). To explore the potential mechanism of coagulase in survival, we examined whether the deletion mutation of *coa* alters the susceptibility to the ROS H_2_O_2_. The deletion mutation of *coa* increased the bacterial survival compared to the 923/pYH4 control strain (68 vs. 53%, Figure 5A), whereas the episomally *coa* complemented strain had significantly decreased survival compared to the *coa* deletion mutant (68 vs. 51%). Similarly, heterologous expression of coagulase reduced the ability of coagulase negative *S. epidermidis* to tolerate H_2_O_2_—mediated killing compared to the *S. epidermidis*/pYH4 control (74 vs. 93%, Figure 5B).

### The involvement of SaeRS in survival of *S. aureus* in human blood is coagulase dependent

**Figure 6:**
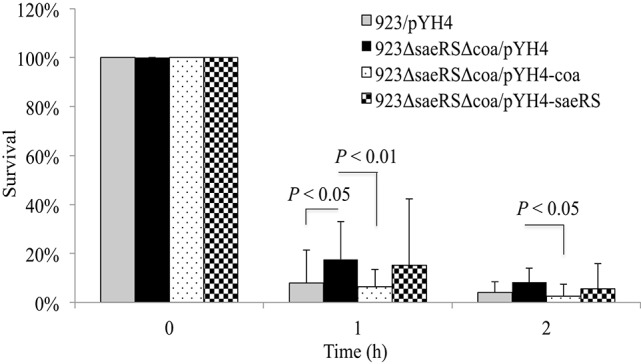
**Effect of ***saeRS*** and ***coa*** double mutation, and complementation on survival of ***S. aureus*** in blood**. Survival of wild-type USA300 CA-MRSA 923, *saeRS*/*coa* double deletion mutant, and *coa* or *saeRS* complementation strain in human blood. Data is the mean and standard error of at least four experiments per strain.

The above studies demonstrated both SaeRS and coagulase are involved in controlling *S. aureus* survival in human blood. To further understand whether the role of SaeRS is attributable to its positive regulation of *coa*, we created *saeRS*/*coa* double mutants in 923 and MW2 strains and examined the effect of expression of either *coa* or *saeRS in trans* on survival of the double mutant in blood. Similar to the results of *coa* or *saeRS* single gene null mutant, the s*aeRS*/*coa* double null mutation in 923 resulted in a significant increase of bacterial survival in the blood (Figure 6). The expression of coagulase *in trans* complemented the survival ability of the *saeRS*/*coa* double mutants to their wild-type control level, whereas the expression of SaeRS *in trans* had no impact on the survival ability of the *saeRS*/*coa* double mutants (Figure 6). Similar results of *saeRS*/*coa* double mutant of MW2 were observed (data not shown).

## Discussion

Our results clearly indicate the roles of SaeRS and coagulase in *S. aureus* survival in human blood. Despite the fact that SaeRS plays a critical role in bacterial invasion of host cells, cytotoxicity, and pathogenicity of *S. aureus* in animal models of infection (Liang et al., 2006; Voyich et al., 2009), it did not appear to enhance survival in human blood. Conversely, SaeRS inhibited the survival of *S. aureus* in human blood, as demonstrated by experiments with the *saeRS* null mutants and by complementation studies. Surprisingly, the expression of *coa in trans* could eliminate the enhanced survival of the SaeRS null mutant in blood, which was demonstrated with *coa* complementation studies in the *saeRS* single and *saeRS*/*coa* double mutants. Consistently, coagulase decreases the bacterial survival in the blood, as supported by experiments with the *coa* null mutants, *coa* heterologous expression strains, recombinant coagulase, as well as by complementation experiments. This finding is at odds with the assumption coagulase could protect bacteria from immune defenses by causing localized clotting. Conversely, our data suggest localized clotting mediated by coagulase may be detrimental to the survival of *S. aureus* in blood. Interestingly, our data indicate that coagulase is somehow associated with *S. aureus* susceptibility to hydrogen peroxide killing.

Our findings in this study only pinpoint the role of SaeRS in control of *S. aureus* survival in human blood through regulation of coagulase production. This suggests that SaeRS may play different roles in different stages of host cell-pathogen interactions. Several key steps of infection include bacterial colonization, proliferation, evasion of innate defenses, and cytotoxicity (Ji et al., 1999; Okumura and Nizet, 2014; Janhsen et al., 2016; McGuinness et al., 2016). It was clearly demonstrated that SaeRS is critical for *S. aureus* to adhere to and invade human epithelial and endothelial cells through regulation of adhesins and invasins (Giraudo et al., 1999; Liang et al., 2006; Sun et al., 2010). Fibronectin-binding proteins (FnBPs) are one kind of major adhesins and invasins, which enable to assemble Fn to connect the host and bacterial cells via binding its receptor α_5_β_1_-integrin (Sinha et al., 1999; Dziewanowska et al., 2000; Fowler et al., 2000). Moreover, SaeRS is crucial for *S. aureus* to mediate the expression of toxins, which are required for cytotoxicity *in vitro* cell culture (Liang et al., 2006; Liang and Ji, 2007) and pathogenicity in different models of infection (Kernodle et al., 1997; Ji et al., 1999; Schwan et al., 2003).

Our results indicate that the SaeRS system controls the capacity of *S. aureus* survival in human blood, which contradicts to previous report that the *saeRS* mutation decreased survival of MW2 strain in human blood (Voyich et al., 2009). To rule out possible effect of genetic background of different *S. aureus* isolates, we utilized the *saeRS* null mutant and its parental control MW2 (which are kindly provided by Dr. Voyich) and conducted survival assays. Consistent with the results of the SaeRS mutants of WCUH29 and 923 isolates, the *saeRS* mutant of MW2 exhibited the similar elevated survival capacity compared to its wild-type control. This contradiction, we believe, is mainly due to the different survival ability of wild type control MW2 in the blood samples. In our studies, <10% of the MW2 cells were able to survive in human blood after 1–3 h; in contrast more than 100% of MW2 cells survived after 3 h of incubation in human blood in the previous report (Voyich et al., 2009). This inconsistence is possibly attributable to the differences in the human blood, as different individuals likely possess different levels of antibodies against *S. aureus*, which would affect the opsonophagocytic killing process (Pier and Elcock, 1984; Chen et al., 2013; Humphries et al., 2015). Another explanation is that in our experiments *S. aureus* from the stationary phase were utilized, while Voyich et al used the mid-exponential phase of bacterial cells. It is possible that the temporal regulation of *coa* by Agr (Novick, 2003) and other regulators that are expressed at different temporal periods may influence the results as the mid-exponential cells grow faster than stationary cells. In addition, it is necessary to further elucidate the influence of antibodies against *S. aureus* in order to define the role of SaeRS in bacterial survival in the blood in future studies.

Regarding coagulase, our results clearly showed that the addition of rCoa to the blood enhanced the susceptibility of *S. aureus* to phagocytic killing. Moreover, altering the expression of coagulase affects bacterial sensitivity to hydrogen peroxide killing. It is necessary to further determine the mechanism in future studies. Our finding is supported with previous reports demonstrating that coagulase is not a virulence factor in several infection models as the *coa* null mutation had no impact on pathogenicity in a rat endocarditis model of infection (Baddour et al., 1994; Moreillon et al., 1995). Moreover, coagulase negative staphylococci (CNS) have become a major nosocomial pathogen (Becker et al., 2014). Additionally, CNS infections cause renal abscesses and lethality in a mouse model of blood stream infection (Wang et al., 2015). On the other hand, our results are inconsistent with recent report that indicated coagulase is a critical virulence factor for abscess formation and bacteremia in a mouse model of blood stream infection (Cheng et al., 2010). This inconsistence is probably due to the difference of animal models of infection and the variation of genetic background for *S. aureus* isolates in these studies. Moreover, in this study we found that unlike 923 and MW2 isolates, the deletion mutation of *coa* could not abolish the coagulation activity of WCUH29, suggesting that other factors, such as von Willebrand factor binding protein, are likely involved in coagulation activity as reported (Thomer et al., 2013).

In conclusion, we identified that the SaeRS two-component system plays an important role in survival of *S. aureus* in human blood via regulation of coagulase production. Coagulase alleviates the survival capacity of *S. aureus* in human blood. These data suggest that coagulase might be used as an alternative strategy to treat *S. aureus*-induced bacteremia.

## Ethics statement

This study was carried out in accordance with the recommendations of NIH guidelines with written informed consent from all subjects. All subjects gave written informed consent in accordance with the Declaration of Helsinki. The protocol was approved by the University of Minnesota Institutional Review Board.

## Author contributions

HG, JH, and JY performed the experiments. HG, JH, and YJ designed the experiments. HG, JH, JY, and YJ analyzed the data. HG, JH, and YJ wrote the manuscript.

### Conflict of interest statement

The authors declare that the research was conducted in the absence of any commercial or financial relationships that could be construed as a potential conflict of interest.
